# Novel Daptomycin Tolerance and Resistance Mutations in Methicillin-Resistant Staphylococcus aureus from Adaptive Laboratory Evolution

**DOI:** 10.1128/mSphere.00692-21

**Published:** 2021-09-29

**Authors:** Jordy Evan Sulaiman, Henry Lam

**Affiliations:** a Department of Chemical and Biological Engineering, The Hong Kong University of Science & Technology, Kowloon, Hong Kong; Antimicrobial Development Specialists, LLC

**Keywords:** MRSA, antibiotics, daptomycin, evolution, tolerance, resistance

## Abstract

It has been shown recently in a number of *in vitro* laboratory evolution experiments that under repetitive antibiotic exposure, bacterial populations can adapt quickly to the treatment condition by becoming tolerant and/or resistant to the drug. The repeated killing and regrowth cycles hasten the selection for tolerant/resistant mutants with survival advantages. Due to the random nature of mutagenesis and the large target size of tolerance mutations, this dynamic evolutionary process appears to be highly unpredictable, generating distinct mutants even under identical, well-controlled laboratory conditions. Here, we utilized an adaptive laboratory evolution (ALE) experiment to hunt for novel tolerance and resistance mutations by subjecting multiple lineages of methicillin-resistant Staphylococcus aureus (MRSA) to repetitive daptomycin treatment. By sequencing multiple isolates along the course of evolution, we obtained three tolerant mutants that have different tolerance levels and identified novel daptomycin resistance mutations in the *mprF* gene. In addition, we found that tolerance/resistance development is more rapid if the population is treated in the exponential phase than if it is treated in the stationary phase, which is likely attributable to the more effective killing of growing cells by the antibiotic. Through competition assays, we found that whether or not the resistant mutants can take over the population heavily depends on the relative survival advantages conferred by the tolerance and resistance mutations. This study reports novel daptomycin resistance and tolerance mutations and offers new insights into the dynamics of the development of tolerance and resistance in bacterial populations under antibiotic exposure.

**IMPORTANCE** Although the phenotype of increased tolerance and/or resistance was commonly observed in evolved populations from typical adaptive laboratory evolution (ALE) experiments, a wide variety of mutations that underlie those phenotypes have been discovered. Therefore, performing ALE experiments in multiple populations in parallel would serve the purpose of mining for different tolerant/resistant mutants and would be useful to explore the diverse population dynamics of evolution. In this study, we performed *in vitro* evolution in a clinically relevant methicillin-resistant Staphylococcus aureus (MRSA) pathogen, using a lethal concentration of a drug that is frequently used in the clinic, daptomycin. Using this strategy, we obtained three distinct daptomycin-tolerant mutants and identified six daptomycin resistance mutations in different locations on the *mprF* gene, collectively adding to our current knowledge of this important pathogen. In addition, we found out that in most cases, the daptomycin-resistant mutant outcompetes other susceptible and tolerant mutants and becomes established in the final population. Follow-up competition experiments offered an explanation; the resistant mutant cannot invade populations of tolerant mutants that confer higher survival advantages than itself. In summary, we demonstrated an experimental strategy to explore the landscape and dynamics of the evolution of tolerance and resistance in MRSA toward daptomycin and made observations that will guide future ALE experiments.

## OBSERVATION

Bacterial populations can cope with and adapt to various stress conditions, including repetitive antibiotic treatment. The two known bacterial adaptation strategies toward antibiotics are resistance and tolerance (1–3). Resistance is more well studied; it directly counters the antibiotic’s action mechanism and allows bacteria to grow at an elevated antibiotic concentration, most often by directly inactivating the drug, altering the drug targets to reduce binding affinity, and decreasing uptake or increasing efflux (4). A resistant population can be detected through an elevation in the MIC, which is the lowest concentration that would kill or inhibit the growth of bacteria (5). Tolerance, on the other hand, describes the ability of a population to survive, but not grow nor replicate, under an extremely high dose of antibiotic for an extended period. Therefore, a tolerant population does not have a difference in the MIC compared to a susceptible population, but they exhibit a slower killing rate. If the tolerance phenotype only occurs in a subpopulation of cells, this is known as persistence (6, 7). Persister cells (the tolerant subpopulation) are present naturally in almost every bacterial population, and it is known to be a phenotypic state rather than a genetic trait (8, 9). It is often interpreted as a bet-hedging strategy of bacteria in order to outlive unfavorable environmental stresses (10).

Through *in vitro* adaptive laboratory evolution (ALE) experiments, several groups have shown that antibiotic tolerance rapidly evolves in the population, leading to significantly higher survival of the antibiotic after only a few cycles of repetitive drug exposure (11–17). Laboratory evolution is proven to be a powerful tool for studying antibiotic tolerance, but most of the studies to date were performed in Gram-negative bacteria (such as the model organism Escherichia coli) and not in a clinically dangerous pathogen such as methicillin-resistant Staphylococcus aureus (MRSA), which is Gram positive. In addition, although it is known that in such laboratory evolution experiments, bacterial populations eventually become tolerant or resistant to the drug after several rounds of antibiotic treatment (18), the evolutionary path leading to the final population genotype is known to be highly variable and unpredictable. That is, the mutations that emerge from the populations that received the same treatment could be different (16). In this study, we performed *in vitro* laboratory evolution in MRSA using one of the most important antibiotics used to treat its infection in clinics, daptomycin (19). Daptomycin is the only cyclic lipopeptide antibiotic that has clinical approval and is active only against Gram-positive bacteria such as S. aureus and several Streptococcus and *Enterococcus* species (20). Although the action mechanism of daptomycin is still debatable, it is proposed that daptomycin disrupts multiple aspects of cell membrane functions (e.g., by binding to the cell membrane and causing rapid membrane depolarization) and inhibits DNA, RNA, and protein synthesis, which eventually leads to cell death (21). Using this experimental strategy, we mined novel daptomycin tolerance and resistance mutants, which were isolated at specific time points during the evolution experiments. In addition, although we observed that in some lineages the final population became tolerant to the drug, in most of the lineages the final population became resistant (without tolerance mutations). Interestingly, in the latter case, tolerant isolates were detected in the middle cycles of the evolution experiments. To elucidate the possible scenarios during the evolution, we performed time-kill assays and conducted competition experiments between the emerging tolerant/resistant mutants. Overall, this study reported novel daptomycin tolerance and resistance mutations and provides a glimpse of how MRSA in different growth phases evolves under repetitive daptomycin treatment.

### Adaptive laboratory evolution of exponential- and stationary-phase MRSA.

**FIG 1:**
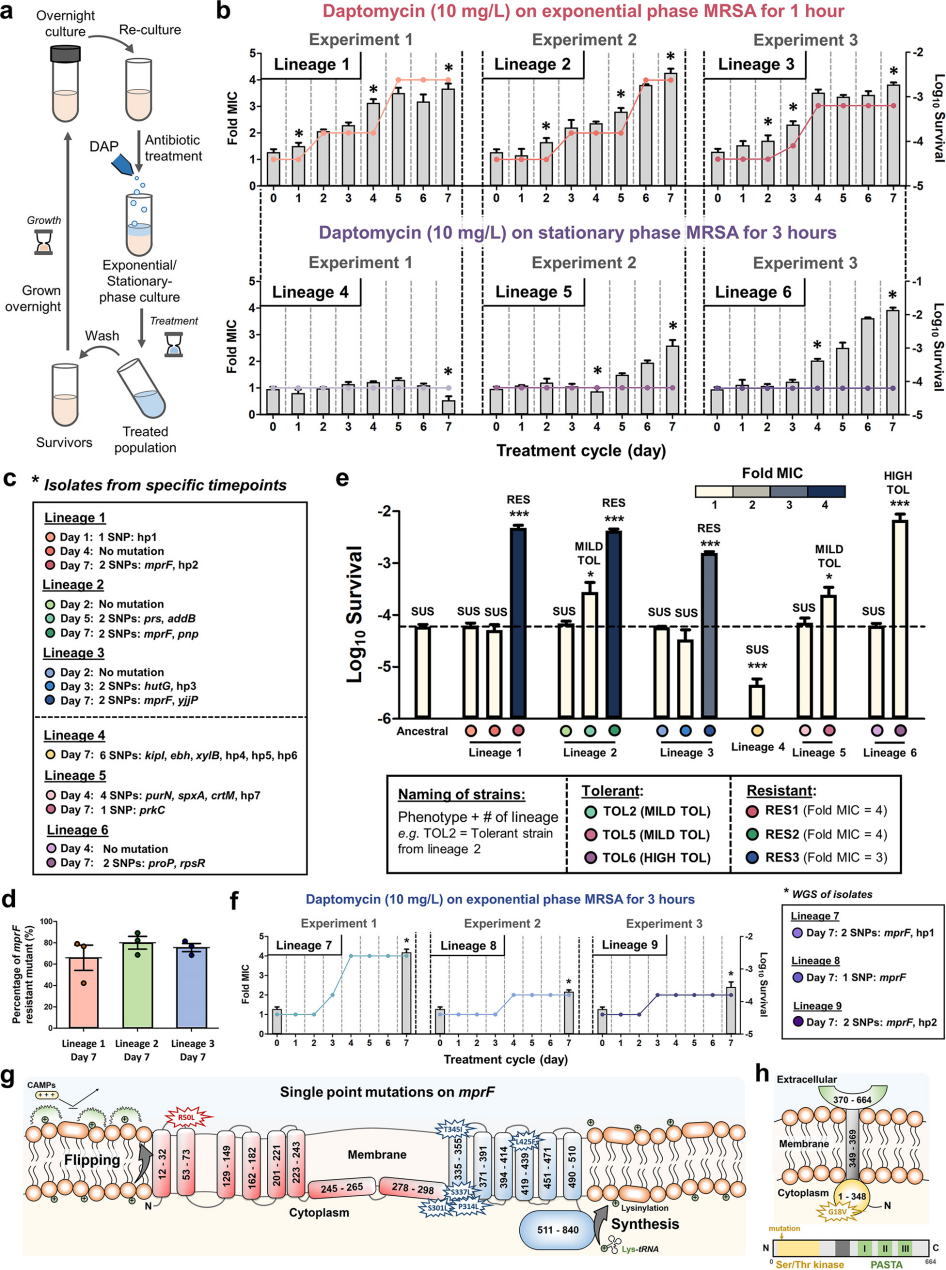
Adaptive laboratory evolution of MRSA. (a) Experimental design of the evolution experiment. Early-exponential-phase or stationary phase MRSA was repetitively treated with daptomycin (10 mg/liter), washed, and regrown overnight. The cycle was repeated for 7 days. (b) Development of tolerance and resistance in early-exponential-phase (top) and early-stationary-phase (bottom) MRSA populations under cyclic daptomycin treatment. Three independent experiments were performed for each exponential- and stationary-phase population, generating a total of 6 lineages. The MIC of the population toward daptomycin (colored dots connected by a line) and the fraction of surviving cells (gray bars) in each cycle are shown (mean ± standard error of the mean [SEM], *n* = 3). Asterisks indicate time points where a random isolate was picked for sequencing. (c) Summary of the nonsynonymous point mutations observed in the isolates from specific time points during the evolution experiment in panel b, marked with asterisks. For each lineage, a random isolate was taken at specific time points (marked by different colored circles) and subjected to whole-genome sequencing. The name of the genes where the mutation occurs was shown (for details, see Table 1). SNP, single point mutation; hp-no., hypothetical protein (the numbers indicate different proteins); SUS, susceptible; TOL, tolerant; RES, resistant. (d) Proportion of *mprF* resistant mutants in the population after 1 week of treatment. Aliquots of the population from lineages 1, 2, and 3 after the seventh day of treatment were serially diluted and plated on agar plates containing daptomycin (1 μg/ml) (to estimate the number of resistant bacteria) and an agar plate without antibiotic (to estimate the total number of bacteria) (mean ± SEM, *n* = 3). (e) Survival of the isolates in panel c after 3 h of daptomycin treatment (10 mg/liter) (mean ± SEM, *n* = 3). The MIC of the isolates are indicated by a gradient of yellow to blue color (more yellow means lower MIC and more blue means higher MIC). From all isolates, 3 were found to be tolerant (TOL2, TOL5, TOL6) and 3 were found to be resistant (RES1, RES2, RES3) toward daptomycin. The horizontal dashed line shows the mean survival of the ancestral population. Significance of difference from the ancestral strain: *, *P < *0.05; ***, *P < *0.001 (two-tailed *t* test). (f) Development of tolerance and resistance in early-exponential-phase MRSA populations under cyclic daptomycin treatment (10 mg/liter) for 3 h. Three independent experiments were performed, generating an additional 3 lineages to the previous evolution experiments. The MIC of the population toward daptomycin in every cycle (colored dots connected by a line) and the fraction of surviving cells at the beginning and the end cycle (gray bars) are indicated (mean ± SEM, *n* = 3). Asterisks indicate time points where a random isolate was picked for sequencing. The right panel shows the summary of nonsynonymous mutations observed in the isolates from the last day of the evolution experiment, as detected through whole-genome sequencing. The names of the genes where the mutation occurs are shown (for details, see Table 1). SNP, single point mutation; hp-no., hypothetical protein (the numbers indicate different proteins). (g) Single point mutations on the *mprF* gene observed in lineages 1 to 3 in (panel b, top) and lineages 7 to 9 in (panel f) (R50L, S337L, L425F, T345I, P314L, and R301L). (g) The *mprF* topology was adopted from a previous study (44), and the *mprF* synthase and flippase domains are shown in blue and red, respectively. (h) Mutation on the *prkC* gene on TOL5 (G18V) which confers mild daptomycin tolerance. The gene consists of a kinase domain (yellow), transmembrane domain (dark gray), and three penicillin and Ser/Thr kinase-Associated (PASTA) repeat sequences (green).

We performed adaptive laboratory evolution (ALE) experiments by repetitively treating methicillin-resistant Staphylococcus aureus (MRSA) with a lethal dose of daptomycin (10 mg/liter, ∼10× MIC) during the exponential and stationary phases (Fig. 1a). After a week of treatment, we observed that resistance was consistently observed in all of the populations that were treated during the exponential phase, with a 3- to 4-fold increase in the MIC (lineages 1 to 3, Fig. 1b, top). (The increase in the MIC surpasses the daptomycin breakpoint of 1 mg/liter for MRSA according to the Clinical and Laboratory Standards Institute [CLSI] [22].) On the other hand, populations treated during the stationary phase do not exhibit a change in the MIC compared to the ancestral strain (lineages 4 to 6), but two of the populations (lineage 5 and 6) have increased survival after a week (Fig. 1b, bottom), suggesting that they are tolerant to the antibiotic.

We sequenced several isolates from specific time points during the evolution experiment (marked by asterisks in Fig. 1b) and detected several single point mutations (marked by circles with different colors in Fig. 1c). While all of the isolates from the exponential-phase-treated populations after a week of treatment have a mutation in the *mprF* gene, which was known to govern daptomycin resistance (23), isolates from the stationary-phase-treated populations bear mutations in different genes unrelated to daptomycin resistance (Table 1). The gene *mprF* codes for phosphatidylglycerol lysyltransferase, which catalyzes the transfer of a lysyl group from l-lysyl-tRNA (Lys) to membrane-bound phosphatidylglycerol (PG), producing the positively charged lysyl-phosphatidylglycerol (LysPG), a major component of the bacterial membrane. Other isolates from the early and middle cycles of the exponential-phase-treated populations (lineages 1 to 3) either have no mutation or possess point mutations in different genes other than *mprF*, suggesting that the mutations in *mprF* appeared in the populations near the end of the evolution experiment. In all of the three lineages where resistance through *mprF* mutation was observed (lineages 1 to 3), the proportion of *mprF* mutant comprises the majority of the population after a week of the evolution experiment, indicating that resistance is already established in the population at this point (Fig. 1d).

**TABLE 1:**
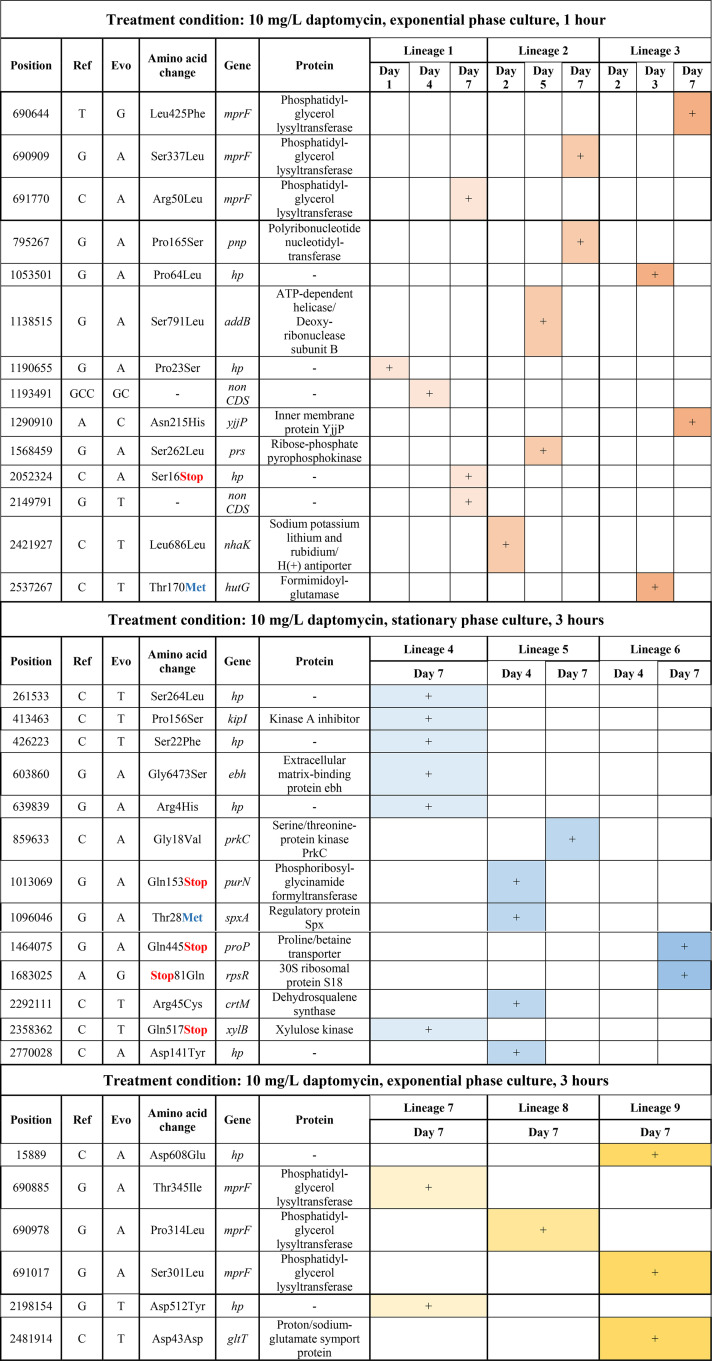
Complete list of mutations detected in the evolved strains from whole-genome sequencing^*a*^

a (Top) List of mutations detected in the evolved strains treated with daptomycin (10 mg/liter) for 1 h during the exponential phase (lineages 1 to 3). (Middle) List of mutations detected in the evolved strains treated with daptomycin (10 mg/liter) for 3 h during the stationary phase (lineages 4 to 6). (Bottom) List of mutations detected in the evolved strains treated with daptomycin (10 mg/liter) for 3 h during the exponential phase (lineages 7 to 9). +, The presence of mutation.

To determine the tolerance and resistance phenotypes of the isolates, we measured their MIC and survival after 3 h of daptomycin treatment (Fig. 1e). As expected, isolates that have a mutation in the *mprF* gene (isolated from the seventh day of lineages 1, 2, and 3, called RES1, RES2, and RES3, respectively) are resistant, shown by the increased MIC and survival toward daptomycin. Interestingly, three isolates are tolerant but not resistant, marked by the increased survival toward daptomycin without a change in the MIC. Two of them, namely, an isolate from the fifth day of lineage 2, which has mutations in the *prs* and *addB* genes (TOL2), and an isolate from the seventh day of lineage 5 with a single point mutation in the *prkC* gene (TOL5), have a mild-tolerance phenotype with an ∼5-fold increase in the survival percentage after 3 h of treatment. The other isolate (from the seventh day of lineage 6) with single point mutations in the *rpsR* and *proP* genes (TOL6) has a high-tolerance phenotype with an over 100-fold increase in the survival percentage after 3 h of treatment.

### Distinct point mutations at *mprF* led to daptomycin resistance and were only observed in the exponential-phase-treated population.

All isolates with 3- to 4-fold increased MIC toward daptomycin (seventh day of lineages 1 to 3) have a single point mutation in the *mprF* gene. Noting that populations treated during the stationary phase (lineages 4 to 6) do not develop resistance even with a treatment duration of 3 h, we wondered if the emergence of the *mprF* mutations would be suppressed with a longer treatment duration. Therefore, we performed another set of evolution experiments on exponential-phase culture but with a longer treatment duration (3 h), while keeping all other conditions the same, generating another 3 new lineages (lineages 7 to 9). Prolonging the treatment duration still led to the establishment of daptomycin resistance after a week (Fig. 1f). Isolates from these 3 lineages at the end of the evolution experiment also harbor single point mutations in the *mprF* gene, although the locations of the mutations are different from those of the previous ones (Table 1). This indicated that under daptomycin treatment, the selection pressure for mutation in this gene is strong in the exponential-phase culture, irrespective of the treatment duration, although the exact location of the mutation varies.

In contrast, no *mprF* mutant was observed in the evolved populations that were treated during the stationary phase. We attribute this observation to the higher extent of killing in the exponential-phase culture compared to the stationary-phase one. At a dose of 10 mg/liter of daptomycin, the exponential-phase culture exhibits a steeper killing slope during the treatment and, consequently, a faster invasion of the resistant mutants during the repetitive treatment cycles (1, 7). As previously reported, while daptomycin is strongly bactericidal against exponential-phase cells, it displays a concentration-dependent bactericidal activity against high-inoculum stationary-phase cells (24).

### Novel daptomycin-tolerant and -resistant strains from the evolution experiments.

Our evolution experiments generated three tolerant strains with mutations in different genes (TOL2, TOL5, TOL6) (Fig. 1e) and six resistant strains with a mutation in the same gene, *mprF*, but in different locations (Fig. 1g). Among the six *mprF* mutations, four have been previously reported in both *in vitro* and clinical isolates (R50L [25], P314L [26–34], S337L [27, 29–39], T345I [28, 29, 31, 35, 40–43]), while two others are novel (R301L, L425F). Although the locations of the mutations appear random, most of them are located at the junction of the flippase domain and synthase domain or in the synthase domain of the protein, suggesting that these are the “hot spots” in the gene to cause daptomycin resistance, a phenomenon which was also observed by Ernst and colleagues (44). In addition, we note that different point mutations led to a different degree of increase in the MIC. Mutation in the extracellular region of the flippase domain (R50L) led to a 4-fold increase in MIC, and three mutations in the transmembrane region of the synthase domain (S337L, T345I, and L425F) led to a 3- to 4-fold increase in MIC, while those that occur on the cytoplasmic region of the synthase domain (R301L, P314L) led to a lower increase in MIC (2-fold).

One of our evolved tolerant strains, TOL5, has a single point mutation in *prkC* that resulted in a mild DAP tolerance phenotype (Fig. 1h). In Bacillus subtilis, PrkC senses peptidoglycan fragments through the penicillin and Ser/Thr kinase-associated (PASTA) repeat sequence, to induce spore germination and signal their exit from dormancy (45). It is also known that PrkC phosphorylates elongation factor G (EF-G) *in vitro* and *in vivo* in B. subtilis, which is important for mRNA and tRNA translocation and was known to play a role in persistence (46). Since S. aureus is a non-spore-forming organism, and the mutation occurs in the catalytic kinase domain, the affected function on the evolved strain is likely not related to spore germination or peptidoglycan recognition. In S. aureus, it was reported that the Δ*prkC* mutant has increased resistance to Triton X-100 and fosfomycin, suggesting that there are some cell wall modifications on the mutant (47). In addition, the mutant also had reduced virulence and pathogenesis in a mouse pyelonephritis model. Several lines of indirect evidence also suggested that *prkC* contributes to S. aureus cell wall synthesis. It is known that in S. aureus, *prkC* phosphorylates the response regulator GraR of the two-component system GraRS, and the phosphorylated GraR increases the expression of the *dlt* operon, thus triggering modifications of cell wall teichoic acids (48). These cell wall modifications could then give them a competitive advantage during DAP exposure.

Another evolved strain that has a mild DAP tolerance phenotype is TOL2, which harbors single point mutations in *prs* and *addB*. A mutation in *prs* governing ampicillin tolerance in E. coli was already previously reported by Fridman et al. (13) and appeared to increase the lag time. It is interesting that a mutation in the same gene in a different species, evolved using a different antibiotic, also led to a similar tolerance phenotype. However, the relationship between the essential *prs* gene and the tolerance phenotype is still unknown and requires further study.

**FIG 2:**
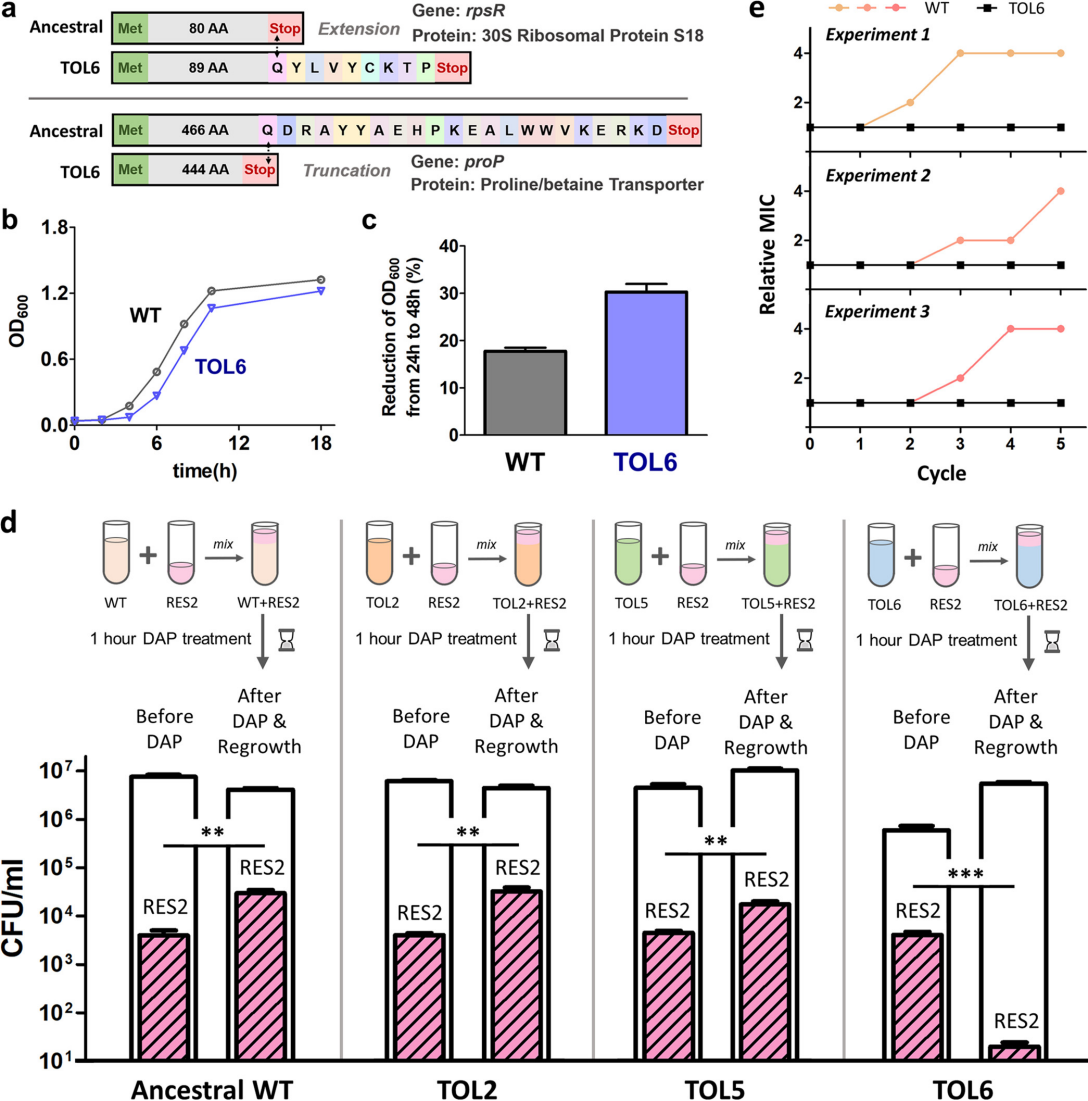
Competition between the daptomycin-resistant strain and tolerant strains determines the final population genotype. (a) A mutation on the *rpsR* (extension of 9 amino acids) and *proP* (truncation of 22 amino acids) gene on TOL6 confers high daptomycin tolerance. (b) Growth curve of TOL6 compared to ancestral WT. The OD_600_ of the cells was measured for 18 h (mean ± SEM, *n* = 3). (c) Reduction in the OD_600_ of TOL6 and ancestral WT from 24 h to 48 h of growth (mean ± SEM, *n* = 3). (d) Competition experiments for daptomycin-susceptible ancestral and tolerant strains (empty bars) with the daptomycin-resistant strain (colored patterned fill). Around 10^3^ to 10^4^ daptomycin-resistant mutants through mutation in *mprF* (RES2) were mixed with 10^6^ to 10^7^ of the wild-type or tolerant strains (ancestral, TOL2, TOL5, or TOL6), treated with DAP (10 μg/ml) for 1 h and then regrown overnight (mean ± SEM, *n* = 3). The number of resistant mutants in the mixed culture after treatment and regrowth was determined by plating in MH agar containing daptomycin (1 μg/ml). Significance of difference with the ancestral strain: **, *P < *0.01; ***, *P < *0.001 (two-tailed *t* test with unequal variances of the log-transformed values). (e) *In vitro* evolution experiments of the wild-type and high-tolerance mutant TOL6 under repetitive antibiotic treatment. Three independent experiments were performed where early-exponential-phase cultures of the WT and TOL6 strain were exposed to 10 μg/ml of daptomycin for 1 h, washed, and regrown overnight. The treatment and regrowth cycles were repeated 5 times. The MIC of the wild-type strain increased to 2- to 4-fold within a few days, but not for the TOL6 strain.

The third tolerant strain, TOL6, has a high-daptomycin-tolerance phenotype, with over 2 orders of magnitude higher survival upon prolonged lethal daptomycin treatment, compared to the ancestral strain. It has single point mutations in the stop codon of *rpsR* and the middle of *proP*, leading to an extension of 9 amino acids and truncation of 22 amino acids, respectively, in the corresponding proteins (Fig. 2a). Since *rpsR* codes for 30S ribosomal protein S18, which is essential for the cells, it suggests that the function of the protein is not abolished in our mutant strain. The gene *proP*, on the other hand, expresses a proline/betaine transporter. Although there was no report directly discussing the relationship between this gene and tolerance, a study examining the transcriptome of a daptomycin-resistant MRSA strain revealed an accumulation of glycine betaine within the cells, coupled with the upregulation of the *cudT* (choline transporter), *betA* (choline dehydrogenase), *gbsA* (glycine betaine aldehyde dehydrogenase), *opuD2*, and *proP* genes (49). In addition, cell wall and membrane-active antibiotics such as daptomycin are known to cause oxidative stress and protein aggregation and misfolding, as revealed by the induction of molecular chaperones (50–52). Glycine betaine gives beneficial effects on protein folding in stressed cells (49, 53–55), where the accumulation of glycine betaine may help the bacteria withstand the stress of antibiotic challenge. Although this mutant has a high tolerance toward daptomycin, the mutations seem to be associated with a high fitness cost under normal growth conditions, as observed from their impaired growth and the increased reduction of cell viability from 24 h to 48 h of growth (during the death phase) (Fig. 2b and c).

### Survival advantages conferred by tolerance and resistance mutations determine the final population genotypes.

We observed mutations that do not appear to cause tolerance or resistance arising randomly in the evolution experiments, which agreed with what is known about bacterial mutations (56). For instance, in lineage 1, a single point mutation in a hypothetical protein was found in the isolate from the first day, but this isolate did not exhibit any increased survival or increased MIC (Fig. 1e). Similarly, in lineage 3, lineage 4, and lineage 5, isolates with no detected tolerance or resistance contain point mutations in a number of genes (a mutation in *hutG* and a hypothetical protein in the isolate from the third day of lineage 3, six single point mutations in different genes in the isolate from the seventh day of lineage 4, and four single point mutations in different genes in the isolate from the fourth day of lineage 5).

This observed random nature of mutagenesis also implies that the development of tolerance should precede that of resistance, at least in most cases. Because a larger set of possible mutations are associated with tolerance compared to that associated with resistance, tolerance mutations should appear more frequently and thus earlier in the evolutionary process (1, 2, 57). Mutations leading to daptomycin resistance are restricted to several genes, such as *mprF* (the most prevalent), *cls*, *walKR*, and the *dlt* operon, which all caused the repulsion of daptomycin from binding to the cell membrane (58), whereas daptomycin-tolerance mutations were observed in a wide range of genes not necessarily directly related to the action mechanism of the antibiotic (those found in this study, such as *prkC*, *prs*, *addB*, *rpsR*, and *proP*, and also those identified in other studies, such as *pgsA* [14], *pitA* [17], *rpoC* and *purR* [59], *snoF*, *hmp1*, *sspA*, and many more [60]). Therefore, in our evolution experiments performed on exponential-phase cultures that eventually led to resistance, we should also expect tolerance mutations to have emerged earlier in the populations.

Indeed, consistent with previous *in vitro* evolution studies in E. coli (18) and Pseudomonas aeruginosa (61) which reported that tolerance precedes resistance, we also observed that a tolerant mutant (TOL2) appeared in an earlier cycle than a resistant mutant (RES2) in lineage 2 (Fig. 1b and c). However, instead of appearing on the background of the tolerant strain, our *mprF* resistance mutation appeared on the ancestral background, meaning that the tolerant mutant did not acquire a new mutation in *mprF*, but the *mprF* resistant mutant outcompeted the tolerant strain and took over the population. As previously mentioned, tolerance mutations are expected to appear earlier owing to the large target size. When the treatment is extended, *mprF* mutations causing daptomycin resistance may appear, but whether it can invade the previously emerging tolerant mutants depends on the survival advantages given by the two mutations during antibiotic treatment. In lineage 2 of our evolution experiment, we observed that the survival advantage conferred by the resistance mutation is higher than that of the tolerance mutation. The TOL2 tolerant mutant has ∼5-fold increased survival, whereas the *mprF* resistant mutant has >50-fold increased survival compared to the ancestral strain (Fig. 1e). In addition, since most tolerance mutations affect the cell’s metabolism, which negatively impacts its growth (62–64), they are expected to incur a higher fitness cost in the absence of antibiotics (compared to the ancestral and resistant mutants). Therefore, once the *mprF* mutation appears on the ancestral background, it should be able to invade the ancestral and tolerant background and take over the population. The fact that we did not observe the resistance mutation appearing on the tolerant background in lineage 2 also implied that the survival advantage or fitness given by both mutations in tandem may be lower than that of the resistance mutation alone.

### The resistant mutant can invade strains with the mild-tolerance phenotype but cannot invade the one with the high-tolerance phenotype.

Although in most cases we observed that the resistant mutant eventually took over the population (lineages 1 to 3, and lineages 7 to 9) and could invade the tolerant mutant (lineage 2), this evolutionary pathway is not always taken. In a previous study, we also observed a situation where a daptomycin tolerance mutation (several base pairs upstream of the *pgsA* gene) that appeared after a week of cyclic daptomycin treatment was retained in the population even after the evolution experiment was extended for 2 weeks, without being replaced by a resistance mutation (14). We rationalized that this is because the survival advantage conferred by the *mprF* resistance mutation is lower than that of the *pgsA* tolerance mutation (the *mprF* mutation led to an ∼40-fold increase in survival, whereas the mutation upstream of *pgsA* led to an over 100-fold increase in survival upon 3 h of 10 mg/liter daptomycin treatment, which was the condition used during the evolution experiment). Therefore, even if a resistant mutant arises, it cannot invade the tolerant mutant. Similarly, in a study by Levin-Reisman et al. (18), in which E. coli is subjected to ALE with ampicillin, the population attained an ampicillin tolerance mutation in *metG*, without being replaced by an *ampC* resistance mutation. This was also explained by the fact that the survival advantage conferred by the *metG* tolerance mutation is almost an order of magnitude higher than that of the *ampC* resistance mutation.

With this insight, we hypothesize that *mprF* resistant mutants (with 25- to 80-fold increases in survival as observed from RES1, RES2, and RES3 in Fig. 1e) cannot invade a high-tolerance mutant such as TOL6 (which has ∼120-fold increased survival; Fig. 1e). To verify our hypothesis, we performed competition experiments where a small number of daptomycin-resistant strain (RES2) organisms were mixed with a larger number of susceptible ancestral wild-type or tolerant strains that confer different survival advantages (TOL2, TOL5, TOL6) and then treated with daptomycin (Fig. 2d) following similar protocols in the literature (14, 15, 59). This experiment mimics a scenario where a daptomycin-resistant strain emerged during our evolution experiment and would tell us whether the resistant mutant can invade the population or not. While the daptomycin-resistant mutant survived with an increased proportion, when it was spiked into the ancestral wild-type and the two tolerant strains with lower survival advantages than those of the resistant strain (TOL2 and TOL5), we observed a significant drop in the number of the resistant mutant in the tolerant strain that confers high survival advantage (TOL6). In addition, prolonging the evolution experiments by repetitively treating the TOL6 strain with daptomycin still did not increase the MIC of the population (3 out of 3 independent experiments), proving the inability of resistant mutants to invade the high-tolerance strain (Fig. 2e). This competition experiment also explains why the TOL2 strain appeared in the middle cycle of lineage 2 (Fig. 1b and c) but was invaded by the RES2 strain, which then became established at the end cycles of the evolution experiment (Fig. 1d). Another interesting observation is that while RES2 can invade TOL5 in the competition experiment, we did not observe any increase in the MIC in lineage 5 even after a week of the evolution experiment (Fig. 1b). It might simply be by random chance that no *mprF* mutation had occurred yet in that lineage, but another explanation could be the aforementioned slower invasion of resistant mutants in the stationary-phase culture during the repetitive treatment cycles.

Overall, by performing adaptive laboratory evolution in different MRSA cultures in parallel, this study reported a novel daptomycin resistance mutation in the *mprF* gene and new daptomycin tolerance mutations in MRSA. We demonstrated that this experimental strategy could be useful to hunt for tolerance/resistance mutations in MRSA, adding to our knowledge about this pathogen. In addition, we showed that the competition between specific tolerance and resistance mutants is key in determining the final genotype of the evolved populations.

### Bacterial strains and growth conditions.

The bacterial strain used for the evolution experiment is methicillin-resistant S. aureus (MRSA) ATCC 43300. Exponential-phase cultures were prepared by incubating a 1:200 diluted overnight culture in cation-adjusted Mueller-Hinton (MH) broth until an optical density at 600 nm (OD_600_) of ∼0.1 was reached at 37°C with shaking. The MH broth used in this study was supplemented with Ca^2+^ to a final concentration of 50 mg/liter to mimic the physiological levels of calcium ions, which is important for the concentration-dependent bactericidal activity of daptomycin (65–67). MH agar was used for colony counts.

### Evolution experiment.

Early-exponential-phase cultures and stationary-phase cultures were prepared by incubating a 1:200 diluted overnight culture in 1 ml cation-adjusted Mueller-Hinton (MH) broth for ∼2 h and 6 h, respectively, at 37°C with shaking at 250 rpm. Three treatment conditions were used for the evolution experiments, and three independent experiments were performed for each condition: (i) early-exponential-phase culture exposed to 10 μg/ml daptomycin for 1 h (lineages 1 to 3), (ii) early-stationary-phase culture exposed to 10 μg/ml daptomycin for 3 h (lineages 4 to 6), (iii) early-exponential-phase culture exposed to 10 μg/ml daptomycin for 3 h (lineages 7 to 9). After the treatment, the antibiotic-containing medium was removed by washing three times in MH broth (10 min centrifugation at 4,500 × *g*), and the cells were resuspended in 1 ml fresh MH and grown overnight at 37°C with shaking at 250 rpm. The concentration of daptomycin was chosen to be similar to those from previous studies (59, 68).

### Tolerance and resistance assay.

The concentration of daptomycin used for treatment was 10 mg/liter. To assess cell viability after antibiotic treatment, the number of survivors were counted by serially diluting cultures in MH broth and plating 100 μl on MH agar and spread plates. The MICs of the population were recorded using the broth macrodilution method. The MIC was determined by incubating ∼5 ·10^5^ exponential-phase bacteria in MH medium overnight with various concentrations of antibiotics. The MIC value was determined as the lowest concentration without growth, according to EUCAST guidelines (69).

### Competition experiment.

To test whether a daptomycin-resistant strain (with the *mprF* mutation) can invade the ancestral wild-type or daptomycin-tolerant population following antibiotic treatment, around 10^3^ to 10^4^ cells of the resistant strain were mixed with around 10^6^ to 10^7^ of the WT or the tolerant strains. Killing assays were subsequently performed on the mixed culture under 10 μg/ml daptomycin treatment for 1 h. The cells were washed twice to remove antibiotics, and the culture was regrown in fresh MH broth overnight at 37°C with shaking. The survival of the daptomycin-resistant mutant was evaluated by plating on MH agar containing daptomycin (1 μg/ml), and the survival of the nonresistant strains (WT and tolerant strains) was evaluated by plating on MH agar without the antibiotic, subtracted by the survival of the resistant mutants. This competition experiment has been used by other groups, and the experimental protocol used here is similar to those from the previous studies (14, 15, 59).

### Genomic extraction and whole-genome sequencing.

Genomic DNA from the ancestral strain and isolates of the evolved populations was extracted using a DNeasy blood and tissue kit (Qiagen) according to the manufacturer’s protocol, with the following lysis buffer: 200 μg/ml lysostaphin solution in 20 mM Tris-HCl, pH 8.0, 2 mM sodium EDTA, and 1.2% Triton X-100. DNA was detected by agarose gel electrophoresis and quantified using a NanoVue Plus spectrophotometer (GE Healthcare). The genomic DNA was sent to BGI for paired-end DNBseq sequencing at a 2 × 150-bp read length and 350-bp insert size. Briefly, a total amount of 1 μg of DNA sample was used as input material for the DNA sample preparation. The DNA sample was fragmented by ultrasound on an E220 instrument (Covaris, Brighton, UK) and selected with an Agencourt AMPure XP-medium kit to an average size of 200 to 400 bp. DNA fragments were end-repaired, 3′ adenylated, and ligated with the adapter. The ligation product with adapters was then amplified, and PCR products were purified with an Agencourt AMPure XP-medium kit. The double-stranded PCR products were then heat denatured and circularized by the splint oligonucleotide sequence. The remaining linear molecule was digested with exonuclease. The single-strand circle DNA (ssCir DNA) was formatted as the final library and sequenced using BGISEQ-500, where the ssCir DNA molecule formed a DNA nanoball (DNB) (70) containing more than 300 copies through rolling-cycle replication. The DNBs were loaded into the patterned nanoarray using high-density DNA nanochip technology. Finally, pair-end 150-bp reads were obtained by combinatorial probe-anchor synthesis (cPAS). Sequencing quality was affirmed using the FastQC algorithm. The sequenced data were filtered, and adapter sequences and low-quality data were removed, resulting in the clean data used for subsequent analysis. Specific processing steps are as follows: remove reads whose low-quality nucleotides (Q-value, ≤12) exceed a certain threshold (50% by default), eliminate reads which contain N nucleotides exceeding a certain threshold (50% by default), eliminate adapter contamination, and finally, filter duplication. The clean bases of each sample are ∼1.3 billion bp, and the clean reads are ∼8.7 million reads for each sample. The whole-genome sequencing (WGS) raw data were submitted and are accessible under NCBI BioProject number PRJNA724993.

### Whole-genome sequencing data analysis.

We performed a genomic comparison between the ancestral and evolved strains and the reference genome. The differences between the strains and the reference were obtained by aligning the sample reads with the reference genome (MRSA ATCC 43300 genome downloaded from the ATCC website, September 2020) using the Burrows-Wheeler Aligner (BWA) mapper V0.7.17 (71). The parameters of BWA were as follows: mem -t 4 -k 32 -M -R. The mapping rate is above 99.13% for all strains. SAMtools V1.9 (72) was used to detect single nucleotide polymorphisms (SNPs) and small indels (<50 bp) with the following parameters: mpileup -m 2 -F 0.002 -d 10000 -u -L 10000, and call –ploidy 1 -mv -Ov. The detected SNPs were further filtered with QUAL > 50 so that the final SNP list contains high-quality SNPs with high confidence. Subsequently, Integrative Genomics Viewer (IGV) (73) was used to view the aligned sequence and perform further analysis on the identified SNPs/indels (e.g., determination of amino acid substitution). To verify our results, Snippy V4.6.0 (74), a rapid haploid variant-calling and core genome alignment software that has a built-in filter to detect high-quality SNPs, was used to reanalyze the whole-genome sequencing data. No difference was found in the identification of SNPs using SAMtools and Snippy.

### Data availability.

The whole-genome sequence data have been deposited in the BioProject database (NCBI) under the accession number PRJNA724993.
